# Reframing wearables in therapy: Co-designing compassion-focused, practical guidance for the use of wearables in acute mental healthcare

**DOI:** 10.1177/20552076261483463

**Published:** 2026-09-22

**Authors:** Charlotte M. van Lotringen, Saskia M. Kelders, Gerben J. Westerhof, Matthijs L. Noordzij

**Affiliations:** 1 Department of Psychology, Health and Technology, 3230 University of Twente, Enschede, Netherlands; 2 Optentia Research Focus Area, North-West University, Vanderbijlpark, South Africa

**Keywords:** wearables, mental healthcare, implementation, compassion, co-design, participatory design

## Abstract

**Objective:**

The integration of wearables, such as smartwatches, into mental healthcare holds promise for supporting self-management through the monitoring of physical activity and rest patterns, yet their implementation in clinical practice remains limited. Aligning with the values of stakeholders in mental healthcare may enhance the implementation of technology. An important value in mental healthcare is compassion; recognizing and wanting to alleviate suffering. Therefore, we co-designed compassion-focused implementation materials for the implementation of smartwatches in treatments.

**Methods:**

We conducted 5 co-design sessions with in total 9 patients and 13 professionals from a department for acute mental healthcare. During the co-design process, we iteratively came to a 3 (format: written manual, infographic, card set) × 2 (framing: efficiency-focused, compassion-focused) design, resulting in six prototypes that systematically combined each format with each framing approach to smartwatch use.

**Results:**

Based on the co-design sessions, a card set has been developed that can be used in group sessions to work compassionately with smartwatches. It integrates the preferences of professionals for accessible and tangible guidance that can be used in treatment sessions.

**Conclusions:**

Co-design methodology helped to develop implementation materials based on the needs and preferences of stakeholders in mental healthcare. Moreover, the focus on compassion during the co-design process and in the final card set could help align the use of technology with values of stakeholders for a more meaningful and successful implementation.

## Introduction

Technological interventions, such as online counseling, digital self-help modules, and wearables, have the potential to improve mental health treatments and increase their accessibility.^1^ However, the actual use and implementation of technology in mental healthcare remain inconsistent, with some digital solutions becoming mainstream while others lag behind.^2^ For instance, videoconferencing has seen significant adoption since the COVID-19 pandemic, whereas more innovative solutions, such as wearable devices, have not yet been systematically integrated into routine care^3^ and are met with doubts and resistance from professionals.^4^

The use of wearable technology, such as smartwatches, is a promising development in mental healthcare. These devices can be applied in two primary ways: first, through passive sensing and prediction. Passive sensing leverages physiological and behavioral signals, such as heart rate variability, physical activity, and sleep patterns, to detect and predict changes in mental health status.^5^ While this approach holds potential for early detection and personalized interventions, its clinical implementation remains limited due to challenges in data interpretation, standardization, and integration into routine care.^5^ Second, a more immediate and evidence-based application is using wearables in a more supportive and behavior-focused manner to facilitate behavioral activation and provide insight into rest and relaxation. Similar passively collected data are used here primarily as feedback tools for self-monitoring, reflection, goal-setting, and discussion during treatment, rather than for algorithmic prediction or diagnosis. This type of application overlaps with two well-established pillars in the treatment of common mental health disorders, namely increasing physical activity and addressing sleep disturbances.^6,7^ Smartwatches offer a low-cost and accessible means of promoting these therapeutic goals by enabling self-monitoring, goal-setting, and feedback mechanisms.^7,8^ Therefore, the current study focuses on the application of wearables for tracking and adjusting behavior such as physical activity and sleep, which is a closer match with current daily practice in mental healthcare.

Previous studies have examined the qualities and potential of smartwatches for these types of goals. For registering sleep or wakefulness, smartwatches offer comparable results to actigraphy (motion registration), although they are less reliable and valid when it comes to registering sleep stages such as REM and deep sleep.^9,10^ Furthermore, an umbrella review of 39 meta-studies with 163,992 participants shows that the use of smartwatches is effective in improving physical activity, and that this effect is clinically relevant and sustainable over time.^8^ Additionally, research indicates that clients in mental healthcare settings are receptive to incorporating smartwatches into their treatment, particularly when they receive helpful feedback about their health and remain in control over the data that they share.^11^

Despite the added value that smartwatches can offer in mental healthcare through facilitating insight into sleep and activity and working towards more concrete goals, their implementation and integration into practice lag behind.^11–13^ Rather than reflecting an inherent limitation of digital tools, this implementation gap highlights challenges in how technologies are integrated into relational and organisational care processes. Mental healthcare clients and professionals experience several barriers to their integration. In general, digital mental health interventions are often seen as impersonal by clients,^14^ and mental health professionals have concerns about technology diminishing the personal and empathic bond.^15,16^ Moreover, they indicate that they experience pressure from managers and insurance companies to use technology for increased efficiency and cost savings, which leads to distrust.^15,17^ Additionally, in-depth interviews with professionals reveal that they perceive a lack of clear and meaningful justification for why and how technology should be integrated into treatment.^18^

In order to integrate technology into daily practice, it helps if its use aligns with the motivations and values of those involved in mental healthcare.^17,19–21^ One of the most fundamental values in this field is compassion, the sensitivity towards suffering and the commitment to alleviate it.^22^ Research has consistently linked the presence of compassion to improved clinical outcomes, stronger therapeutic alliances, and enhanced patient engagement.^23–26^ However, compassion is often seen as a purely human emotional response, leading to skepticism about how digital tools can support it.^27^ A helpful, more process-based definition of compassion is proposed by Strauss et al.,^28^ breaking it down into five interrelated components: (1) recognizing suffering, (2) understanding its universality, (3) empathizing with the sufferer, (4) tolerating distress, and (5) acting to alleviate suffering.

This structured framework provides a concise and impactful way to consider both the why and how of technology implementation in mental healthcare.^29^ The why of technology implementation would be to better alleviate human suffering, instead of merely to optimize efficiency. Furthermore, technology could have diverse potential benefits and disadvantages, but how technology is implemented in practice influences the way it is actually used and perceived. Therefore, the how is equally important: implementation strategies should be guided by the same compassion-based processes that define human caregiving.^30^ Although digital technologies are sometimes feared to make care more impersonal or distant, technologies such as smartwatches may also support compassionate mental healthcare when implemented thoughtfully.^29^ For example, wearable data may help clinicians gain a more continuous and contextual understanding of patients’ daily experiences, potentially strengthening empathy, shared understanding, and personalized support.

However, limited work currently exists concerning how smartwatches can best be implemented in mental healthcare,^11,13,31^ let alone how this can connect with compassion. On the one hand, smartwatches could provide real-time insights into sleep and activity, support shared decision-making and goal setting, and potentially improve the therapeutic relationship and compassion.^13,29,32^ On the other hand, monitoring can lead to concerns about one’s own health, an excessive focus on data and its (in)accuracy, and a sense of failure if certain goals are not achieved.^13,16^ Furthermore, smartwatches often focus on aggregated data, and not on a holistic picture of the patient.^12^ In order to achieve the positive effects of monitoring and to prevent or reduce the negative ones, nuanced feedback and explanations of smartwatch-data are recommended.^13^ Thus, providing guidance in the use of smartwatches in treatment is of importance. However, support for professionals in using smartwatches in treatments, and digital mental health interventions in general, is lacking.^18,33^ Typically, studies investigating digital mental health interventions do not take their integration in daily practice into account.^11,29^

Previous research calls for embedding technologies in treatment using co-design with professionals and patients,^11^ in this context referred to as ‘lived experts’.^34^ Co-design involves active collaboration between different stakeholders to jointly design tailored solutions for a specific problem.^35^ Given the findings outlined above, two needs seem to emerge: first, professionals require a meaningful rationale for using wearable technology, one that aligns with the importance of interpersonal, compassionate contact in their work. Second, they need practical guidance on how smartwatches can be integrated into treatment without undermining the therapeutic relationship.

Thus, the aim of our study was to co-design implementation materials for the use of wearables that answer to these needs, together with professionals and lived experts. As mental health professionals act as gatekeepers for the use of technology in care,^36^ and guidance on how to use smartwatches in treatment is lacking, we will focus on materials that support the professional in offering suitable guidance in the use of smartwatches in care, and mainly explore their perspectives here. Through several co-design activities, we collected (1) requirements for and (2) the final design of implementation materials to aid the use of smartwatches in acute mental healthcare. As part of the requirement-gathering process, we presented mental health professionals with both compassion-focused and efficiency-focused materials, in order to explore their preferences and responses to these contrasting implementation framings. Based on the results, we will reflect on how value-based co-design contributes to meaningful and effective implementation strategies.

## Methods

### Design

The current study used qualitative methods and was based on the Guideline for User Participation,^37^ which uses the phases of Design Thinking as a framework. The five phases of Design Thinking are empathizing with users, defining challenges and needs, ideating solutions for the challenges and needs, prototyping, and testing the refined prototype. We conducted a preparatory contextual inquiry and five co-design sessions, each with different activities (see Table 1). The design of the final implementation materials was based on the co-design sessions, and took place in three iterative cycles in-between and after the co-design sessions. During the final iteration, we worked together with a design company to refine the materials and layout of the prototype.

**Table 1.** Overview of the co-design process.

​	Design thinking phase	Activities	Aim	Participants
Contextual inquiry	Empathise	Reading treatment documents, visiting the department, following along with a day of treatment	Familiarisation with the context	​
Session 1	Empathise, define	Lived experts’ treatment journey, compassion needs, smartwatch opportunities and barriers	Understanding treatment and compassion experiences of lived experts, and their view on smartwatch use	2 lived experts
Session 2	Empathise, define, ideate	Professional treatment journey, compassion needs, smartwatch barriers and solutions	Understanding treatment and compassion experiences of professionals, and their view on smartwatch use	6 professionals
Session 3	Ideate, prototype	Evaluating and ranking prototypes with compassion-focus or efficiency-focus, iterating preferred prototypes	Iterating prototypes, gathering preferences for focus of the prototypes	8 professionals
Iteration 1				
Session 4	Prototype, test	Evaluating iterated prototype, testing it in a role play	Prototype evaluation and iteration	2 professionals
Iteration 2				
Session 5	Test	Testing the prototype during a treatment session and collecting feedback	Prototype evaluation and iteration	7 lived experts, 1 professional
Iteration 3 with design company				

### Participants

In total, 13 professionals and 9 lived experts participated in one or more of the 5 co-design sessions. Professionals were 8 women and 5 men with a mean age of 32 (std. dev. 8.6). Professionals were mostly social psychiatric nurses (n=4) or nurses (n=3). Other participating professionals had the following function titles: nurse and social worker (n=1), medical doctor (n=1), social psychiatric nurse in training (n=1), social worker (n=1), nurse specialist in training (n=1), and psychiatrist (n=1). Lived experts were 6 women and 3 men, with a mean age of 43 (std. dev. 11.3). They were in acute mental healthcare for diverse, self-described reasons: bipolar disorder (n=2), depression (n=1), anxiety disorder and depression (n=1), trauma (n=1), autism spectrum disorder and psychosis (n=1), and psychosis (n=1). Two lived experts did not wish to disclose their reason for being in treatment. Participants were recruited via convenience sampling. They received no compensation for participating. Ethical approval was granted by the ethics committee of the faculty of Behavioural, Management and Social Sciences of the University of Twente (registration number: 231067) and by the internal scientific research committee of the involved mental healthcare organization (registration number CWO/CvR24012022). All participants signed written informed consent forms before participating.

### Setting

This study took place within a large mental healthcare organization in the east of the Netherlands. Specifically, professionals and lived experts were involved in Intensive Home Treatment (IHT). This treatment is aimed at people with serious psychiatric problems where regular care is temporarily insufficient and more intensive treatment is required, such as in the case of psychosis, serious depressive complaints, or trauma. The treatment that is offered is outpatient and part-time, takes place every working day, is followed in a group, and lasts an average of 6 weeks. Patients can join the group at any time, and often do not join every working day. Part-time treatment consists of different types of session blocks, such as psychomotor therapy, crisis skills, and psychoeducation. These blocks are usually given by social psychiatric nurses. One of these blocks is ‘Lifestyle’, and this is the first, fixed block in the part-time treatment every weekday morning. This is also the block in which the department aims to use smartwatches. The motivation for using smartwatches is to gain a better picture of the sleep and activity levels of patients and to be able to better respond to this. At the study start, the team had ten smartwatches in its possession, which were only used sporadically by participating patients. Apart from material on how to pair the smartwatch with a smartphone and set it up, there was no material available on how the smartwatches could be useful in treatment, either for professionals or patients.

### Procedure and materials

All sessions lasted between 60 and 90 minutes (mean=74 minutes) and took place between July 17^th^, 2023 and April 12^th^, 2024. Sessions were audio recorded, transcribed verbatim, and anonymized. Unfortunately, the recording of the second session failed, so that only the session materials and extensive notes are available. At the start of each session, participants signed written informed consent forms and demographics were collected. Then, the researcher introduced themselves and the aim of the session. Each session, the researcher emphasized that all input was welcome and helpful, and that there were no right or wrong answers. In all sessions, participants received pens and post-its in different colors.

Before describing the procedure and materials used in each co-design session, we first clarify the distinction between a *compassion provider* and a *compassion receiver*, as we used materials from both of these perspectives. Compassion in mental healthcare is an interactive process involving both a compassion provider and a compassion receiver. A compassion provider, for instance a mental health professional, recognizes suffering and takes action to alleviate it, demonstrating attunement, emotional regulation, and caregiving behaviors.^28,30^ A compassion receiver, such as a client or patient, is the individual experiencing distress and benefitting from acts of compassion, such as emotional support, validation, and concrete assistance in reducing suffering.^38^ Importantly, compassion is not limited to kindness or warmth: it can involve challenging conversations, boundary-setting, and navigating complex power dynamics in care, all in service of reducing suffering by offering and experiencing support in a context-sensitive and meaningful way.^28^

#### Session 1

In the first session, lived experts were each given a large paper (A2 size) with a representation of the timeline of the acute part-time treatment (see Figure 1). Five moments were shown on the timeline: the intake, an average day in the treatment on location, a halfway evaluation meeting, an average day at home during the treatment, and the concluding meeting. Lived experts also received small cut outs of emoticons showing 8 different (emotional) states (excited, happy, neutral, sad, unhappy, scared, angry, and tired). They were asked to select emoticons for different moments on the timeline that matched with their experiences, and to elaborate on their choices and experiences. Next, the researcher explained the concept of compassion in simplified terms (based on^28^). Lived experts received cut outs of visualizations of the five compassion elements from a compassion receiver perspective, with a simplified description (see Figure 2). They were asked to stick them on the timeline if they experienced them in certain moments. Again, they were asked to elaborate on their choices and experiences.

**Figure 1.**
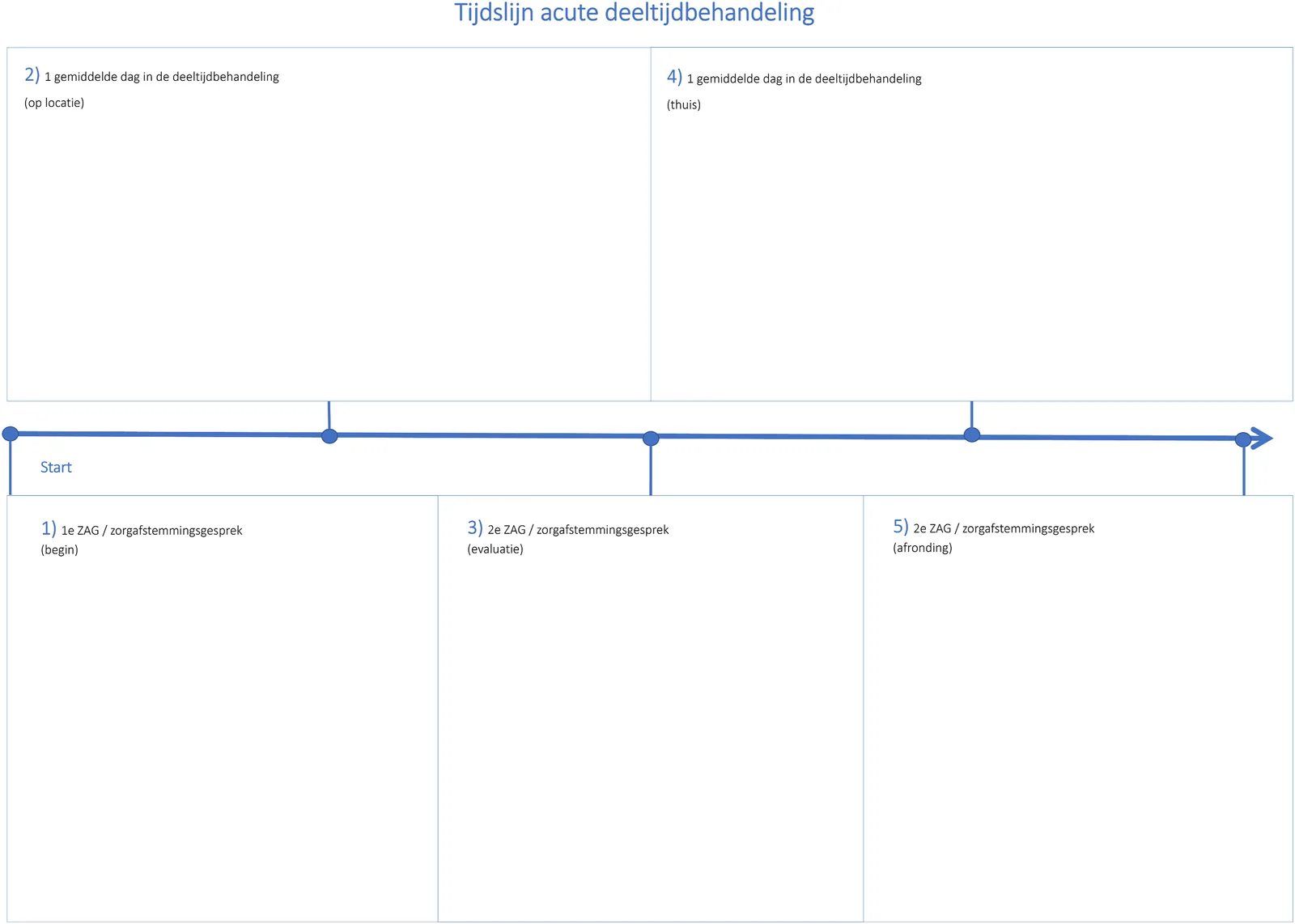
Schematic timeline of the acute part-time treatment showing five moments in the treatment.

**Figure 2.**
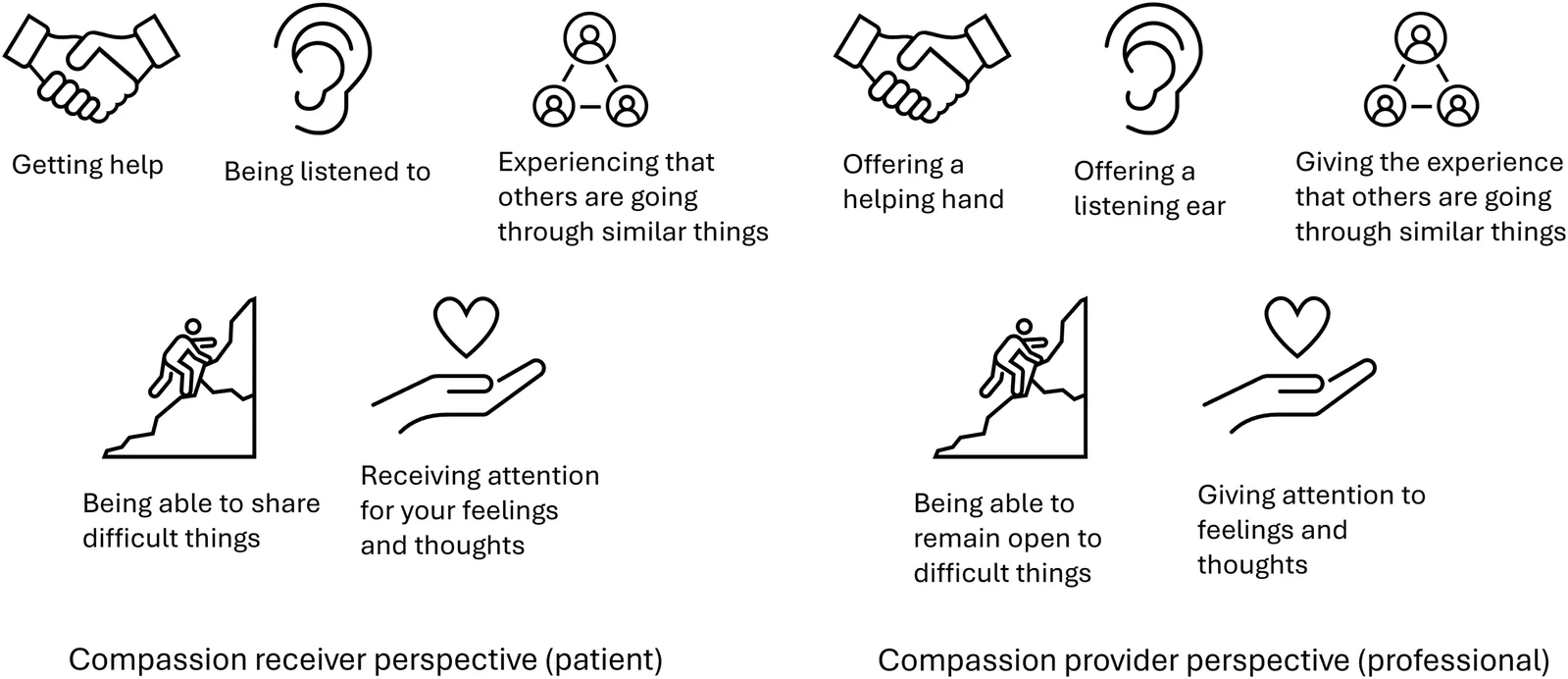
Symbols with simplified descriptions of the five elements of compassion proposed by Strauss et al.^28^ from a compassion receiver perspective (patient, left) and a compassion provider perspective (professional, right). *Note: text has been translated from Dutch to English for the purpose of this paper*.

Finally, the researcher gave a brief introduction on smartwatches and what functions they have (tracking sleep and activity). Lived experts were given cut outs showing the smartwatch that is available at the department (Garmin Vívosmart 4) to stick on the timeline at moments when they saw potential for the use of smartwatches, and they were asked what they thought about the use of smartwatches in treatment. During the whole session, lived experts were also encouraged to use pens or post-its to add their thoughts.

#### Session 2

In session 2, professionals were given the same representation of the timeline of the treatment that lived experts received. They also received visualizations of the compassion elements from a compassion-provider perspective with a simplified description (see Figure 2). They were asked to stick the elements of compassion on the moments on the timeline when they were most important to provide to patients, as well as to add notes on the most important activities during the treatment timeline.

Then, professionals were asked openly about their experiences with the smartwatches. Next, the researcher gave a brief explanation of the smartwatch supported by a PowerPoint (basic functions, evidence base, opportunities other professionals see), showing a smartwatch and the accompanying phone application. Participants received post-its, and were asked to think a few minutes for themselves and to write down barriers for the use of the smartwatches, and what would be needed to minimize these barriers, if possible. Then, these post-its were collected, discussed, and clustered plenary.

#### Session 3

Based on the previous two sessions, 3×2=6 different prototypes were developed: there were three different formats each presented in two different narrative styles. The three formats were a classic written manual (Figure 3), a more visual infographic poster (Figure 4), and a tangible card set (Figure 5). A written manual is closest to the current status quo of protocolized working in mental healthcare, although professionals do also deviate from treatment manuals.^39^ Infographics can be effective to disseminate health-related information in accessible ways.^40^ In contrast to the other two formats, a card-set can offer the needed ‘in the moment’ support for the use of wearables during a treatment session.^41^ All prototypes had two versions: one version with a more practical and goal-oriented style (e.g. main focus on quantitative results and improving efficiency), and one with a more compassionate and process-oriented style (e.g. attention for qualitative experiences, symbols of compassion elements).

**Figure 3.**
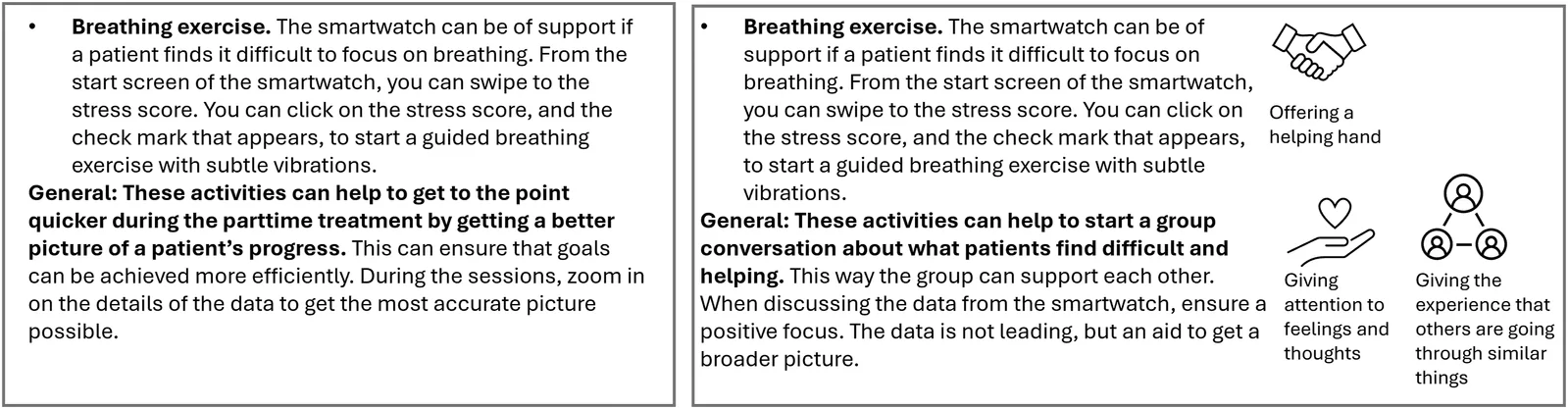
Impression of the written manual prototype in an efficiency-focused (left) and compassion-focused (right) version. The efficiency-focused version described the use of smartwatches to improve treatment efficiency, and prioritized quantitative data and achievements. The compassion-focused version described the use of smartwatches as an aid to get a broader picture of a patient’s situation and to support treatment, and prioritized attention for personal processes. It also contained symbols with descriptions of compassion elements. *Note: text has been translated from Dutch to English for the purpose of this paper*.

**Figure 4.**
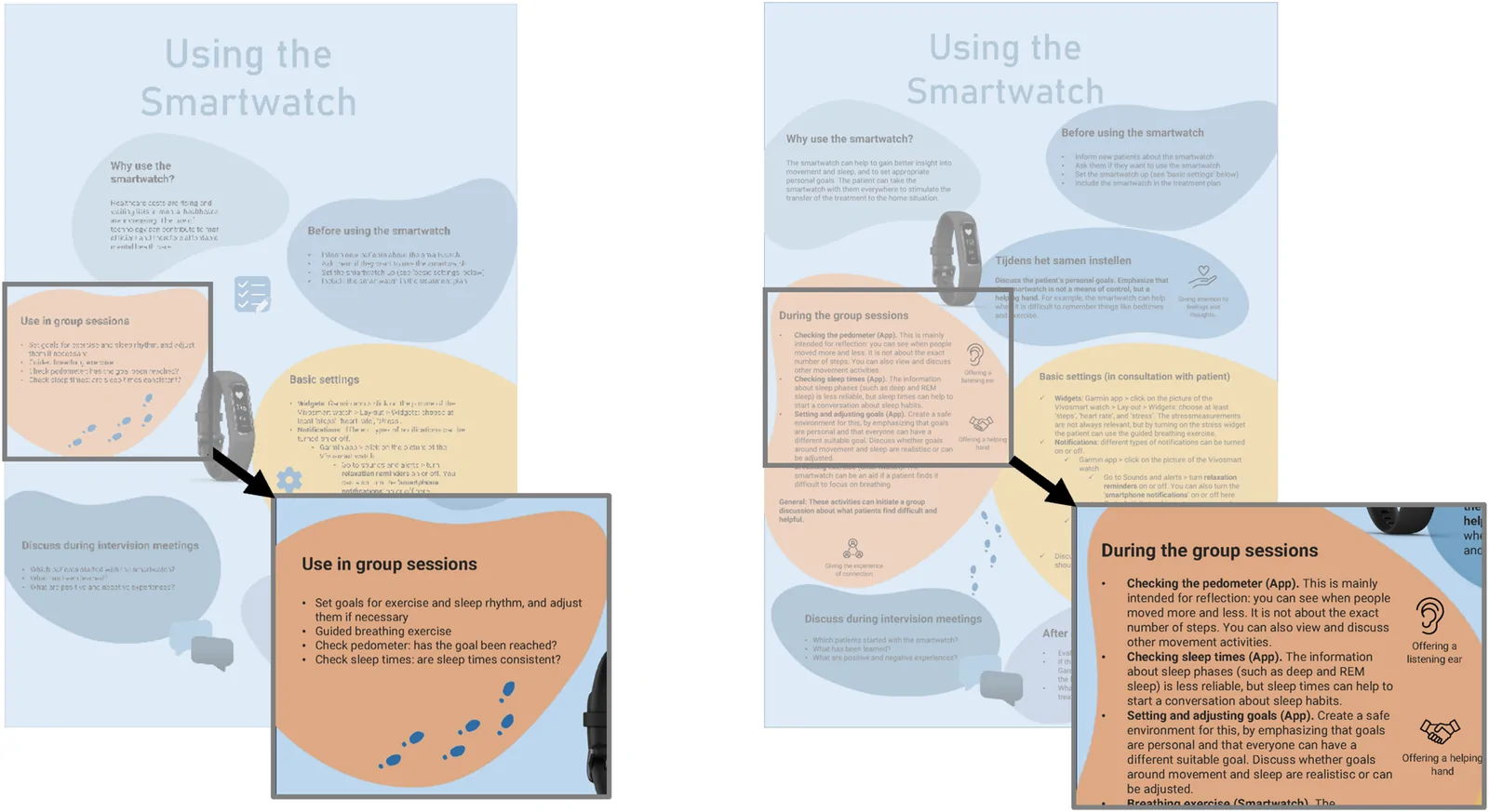
Impression of the infographic prototype in an efficiency-focused (left) and compassion-focused version (right). The efficiency-focused version was straightforward and goal-oriented. The compassion-focused version provided more nuance and focus on the personal process, and contained symbols with descriptions of compassion elements. *Note: text has been translated from Dutch to English for the purpose of this paper*.

**Figure 5.**
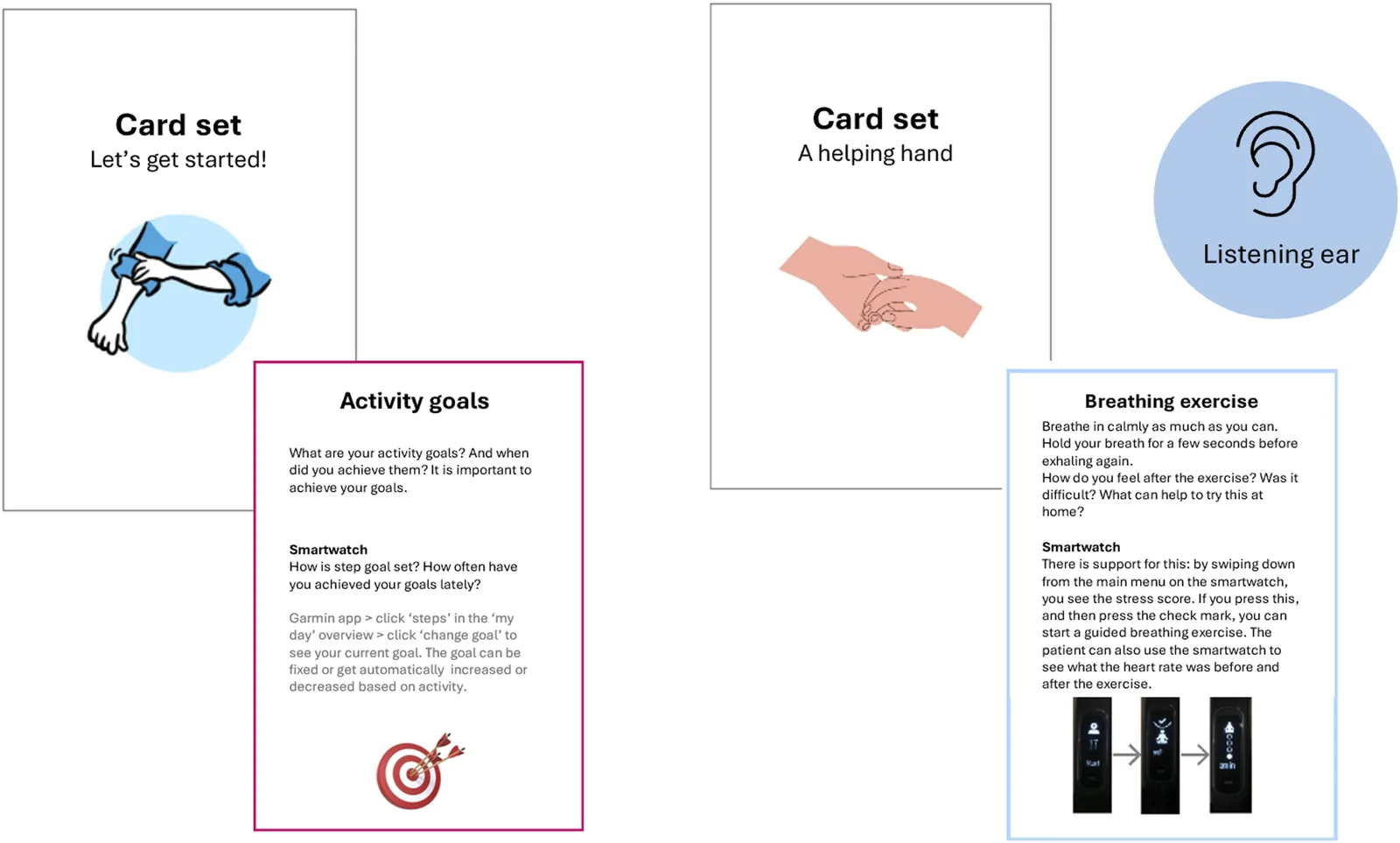
Impression of the card-set prototype used in session 3 in an efficiency-focused (left, title: ‘Let’s get started!’) and compassion-focused (right, title: ‘A helping hand’) version. The efficiency-focused card set was straightforward and goal-oriented. The compassion-focused card set paid more attention to subjective experiences and offered nuance to the quantitative data. It also had compassion symbols on the backs of the cards. *Note: text has been translated from Dutch to English for the purpose of this paper*.

The prototypes were printed on paper and shown in an enlarged and regular size. They were introduced one by one, and handed out to the professionals to examine them. For each prototype, the participants were asked for their reactions. Participants were asked whether they noticed a difference between the two narrative styles, what the difference was according to them and what they thought of both styles. Next, participants were each given a ranking sheet where they could indicate their top 3 of prototypes along with commentary. They were asked to individually rank the prototypes, and add their written comments. These rankings were discussed plenary. Then, participants were divided into duos, and each duo received an enlarged paper version of one of the prototypes, depending on their preference. The duos were asked for their ideas to improve it by writing their comments and ideas on the paper prototype.

#### Session 4

As the majority of participants (6/8) preferred the card set format, a further iteration of the card set prototype (Figure 6) was used in session 4. The card set prototype had cards in different categories (sleep, activity, crisis skills, and general) and the cards had a mix of more ‘to-the point’ and ‘compassionate’ language, based on previously expressed preferences of professionals. Some cards of the prototype were left empty. Furthermore, the written manual was the preferred format of two out of 8 professionals, and four out of 8 professionals were interested in combining the card set with a written manual. Therefore, a prototype of a written manual was offered together with the card set to provide more background information on the use of the smartwatch and its relation to compassionate care. The card set prototype was tested out in a role play with professionals. One participant played the role of the professional leading a session, and the other participant and two researchers (CvL and a master student in Health Sciences) played the role of patients. Both researchers were wearing a smartwatch (Garmin Vívosmart 4). Before the roleplay, the participant with the role of professional had a few minutes to familiarize themselves with the card set. Explicit verbal instructions about the use of the card set were not given, to see the participants’ first impressions and reactions, and to check if the small instruction card of the card set was sufficient to start using the card set. After the role play, there was a discussion about how the card set was experienced both from the professional role and the patient role, what participants noticed, and how the card set could be improved. Then, participants individually wrote positive and negative points about the card set on post-its, which were collected and then briefly discussed. Participants received enlarged copies of the full card set to make notes on improvement points in their own time, and these were later shared with the researchers via email.

**Figure 6.**
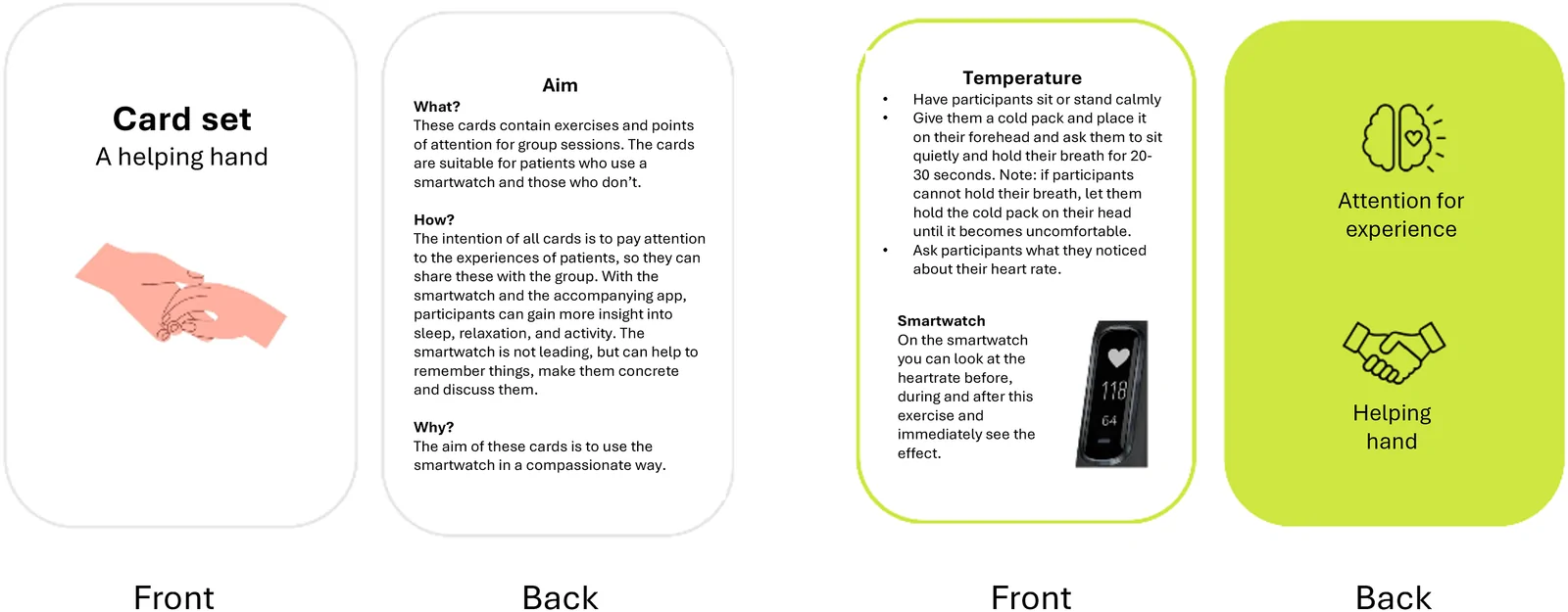
Impression of the iterated card-set prototype used in session 4. The colors on the back of the cards indicate different categories of cards. We used bullet-points and more straightforward text, combined with attention for subjective experiences and compassion symbols.

#### Session 5

Finally, session 5 took place during the first part of a regular ‘lifestyle’ session of the acute parttime treatment. A further iteration of the card set prototype with a written manual was used (Figure 7). Beforehand, the mental health professional giving the session was given a few minutes to familiarize themselves with the card set, and was asked to use the card set in the first part of the session. The researcher was present in this session. The professional introduced the card set in the session, asking a question from one of the cards and opening a group discussion. After all lived experts had taken part in the discussion, the card set was put away, the researcher left the session, and the session continued as usual. After the session, the professional left and the lived experts had a short break. Following the break, the researcher came back and discussed the use of the card set with them. First, lived experts were asked some prompting questions based on the Helpful Aspects of Therapy Form^42^: what was most helpful or important for them personally, and was there anything in the session that might not be helpful? This conversation naturally included a discussion of the use of the smartwatches and the card set. Afterwards, the professional was given a short questionnaire (see Supplemental Material S3) containing questions on the contribution of the smartwatch to compassion (Compassionate Technology Scale for Professionals^43^), questions on the usability and usefulness of the card set: the UMUX lite,^44^ two questions on perceived usefulness based on the Technology Acceptance Model,^45^ one question from the System Usability Scale,^46^ a question on the product-market fit^47^ and a Likelihood to Recommend question.^48^

**Figure 7.**
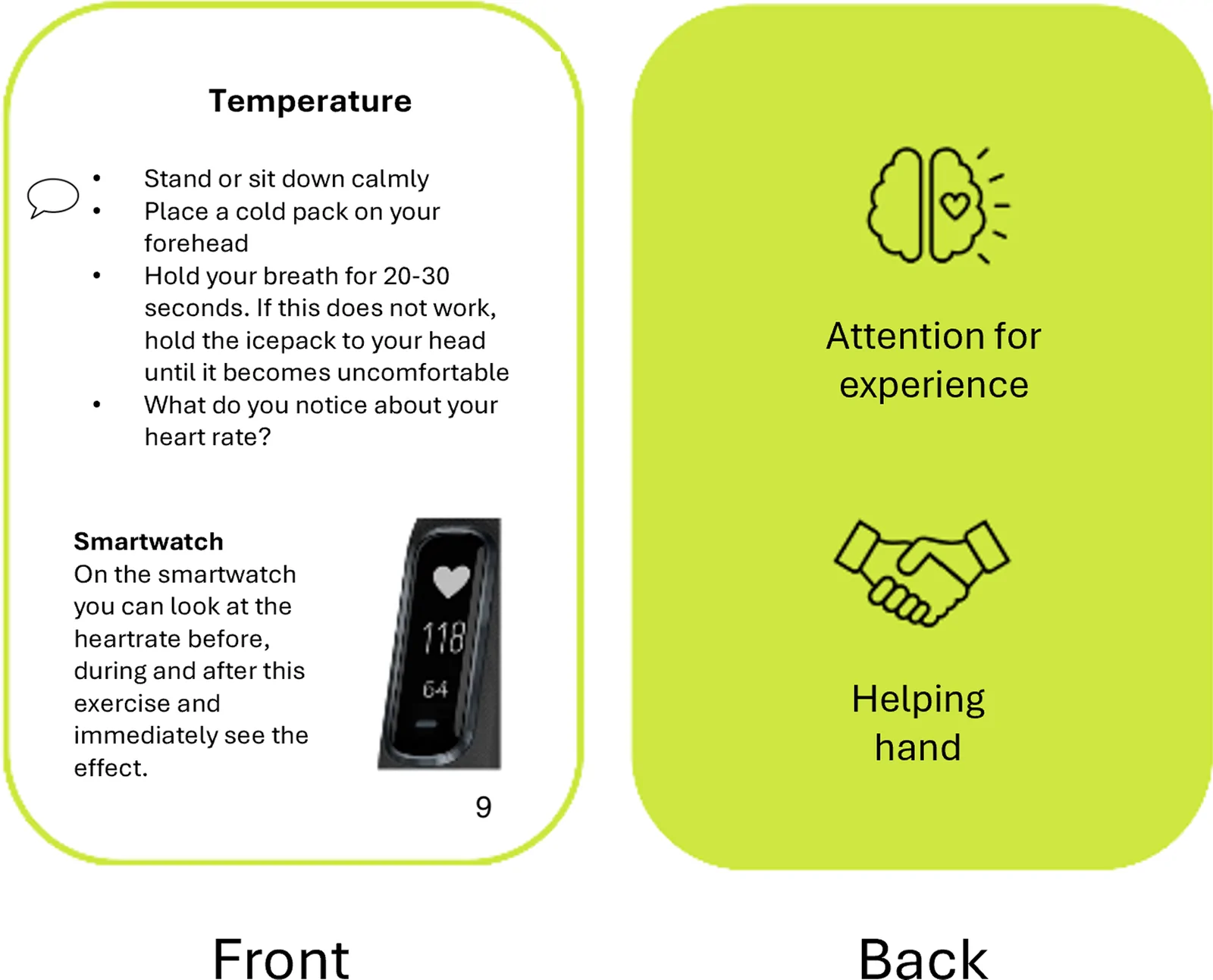
Impression of the iterated card-set prototype used in session 5. Text was made more concise while retaining a focus on personal experiences. Cards were numbered. A speech bubble was added to indicate which text could be used to guide a discussion or exercise in the group.

#### Final iteration with design company

With the help of a design company, we further improved the card set prototype. A summary of the co-design sessions and the collected needs, issues, and requirements, as well as the prototype used in session 5 were shared with a game designer with experience designing for the mental healthcare context and a Bachelor of Science in game design and development. An enduring challenge that was identified in session 4 and 5 was that the group treatment process needed to be protected. The implementation materials had to stimulate relevant, group-based activities and discussions rather than an individualized approach where an individual wearing a smartwatch would get a disproportionate amount of attention at the cost of other patients. Furthermore, participants expressed a wish for a visually pleasing design, and fantasized about cards that patients could take home. Therefore, the designer worked on making the prototype more suitable for the group process, designing take-home cards, and creating a more appealing design with the help of a graphic designer from the same design company. The design process took place in constant consultation between the game designer and the research team.

### Data analysis

The anonymized transcripts, materials, and notes from all co-design sessions were analyzed using a pragmatic qualitative approach aimed at identifying user needs, barriers, suggestions, and design requirements relevant to the implementation of smartwatches in mental healthcare. The purpose of the analysis was to generate practical insights that could inform the development and refinements of implementation materials rather than to develop an explanatory theory.

After each session, the first author (CvL) reviewed the complete transcript together with the accompanying session materials and notes. The unit of analysis consisted of meaningful text segments expressing participants’ opinions, experiences, needs, issues, or requirements regarding treatment and the use and implementation of smartwatches in mental healthcare. These segments were highlighted, and iteratively synthesized into overarching needs and issues across sessions. This synthesis served two purposes. First, it provided a contextual understanding of participants’ experiences and perspectives on treatment, smartwatch use, and compassion. Second, the identified needs and issues were translated into concrete design requirements describing how implementation materials could address identified barriers, support desired practices, or incorporate participant suggestions. The resulting design requirements informed (revisions to) the prototypes before subsequent co-design sessions.

At the start of each subsequent session, the main findings and design decisions resulting from the previous session were summarized and discussed with participants. This provided an opportunity to check whether the researchers’ interpretations reflected participants’ perspectives and to identify missing or misinterpreted issues. Throughout the iterative analysis and design process, the identified needs, issues, design requirements and prototype revisions were regularly discussed within the multidisciplinary research team (CvL, SMK, GLW, MLN), which included expertise in eHealth, compassion, and qualitative research. These discussions supported interpretations of the findings, refinement of design requirements, and reflexivity through critical reflection on the perspectives and assumptions within the research team and how they influenced the analysis and resulting design decisions.

Furthermore, in session 5, the professional who tested the prototype during a group session completed a brief evaluation questionnaire. Responses to this questionnaire were used descriptively as a check for major usability issues that might require immediate revisions to the prototype rather than as a formal quantitate evaluation.

In reporting this study, the standards for reporting qualitative research (SRQR) checklist^49^ was followed (see Supplemental Material S1), as well as the consolidated criteria for reporting qualitative research (COREQ)^50^ (see Supplemental Material S2).

### Researcher reflexivity

CvL conducted the co-design sessions and analyzed them in close collaboration with SMK, GLW, and MLN. CvL had no prior therapeutic, supervisory, or personal relationship with the participants before the study. Participants were aware that the current study was conducted as part of CvL’s PhD research. CvL is a Dutch woman born in the Netherlands and raised in a non-religious environment. She holds a Master’s degree in Psychology and has previous experience with conducting and analyzing qualitative research. She has a strong interest in the mental healthcare context and how mental health professionals and clients experience and shape their treatments. Compassion is a key value to her in personal life as well. While conducting the co-design sessions, she strived for an open and non-prescriptive tone to create space for the stories and perspectives of participants. Compassionate technology for mental health care is the topic of CvL’s PhD research project, supervised by MLN, SMK and GLW. The aim of this PhD project was to investigate how technology in mental healthcare can be integrated into daily practice, where compassion is a fundamental value. This project took place in collaboration with researchers from different disciplines, a mental health care organization, and an eHealth company.

## Results

We will first give a brief overview to contextualize our results, and then describe the outcomes of our study in two parts: (1) the collected requirements for implementation materials, and (2) the final design of a compassion-focused card set to work with wearables, including if and how the requirements were implemented in the design.

### Contextual overview and the value of compassion

From the perspective of lived experts, experiences of care were closely intertwined with fluctuating emotional states, particularly feelings of sadness, anxiety, and uncertainty in the early phases of treatment. Lived experts generally experienced the part-time treatment as a safe place: a ‘safety net’ during an emotionally intense and difficult time. This was in contrast with the emotional strain experienced at home, where understanding and space for expressing difficult emotions were sometimes limited despite supportive intentions. They valued sharing their stories, being listened to, and feeling space for their difficult emotions.

Lived experts also reflected on the use of smartwatches in treatment. While the devices were seen as potentially helpful for increasing insight, motivation, and shared goal-setting, concerns were raised about data accuracy and the potential for misinterpretation. Participants noted that functions such as stress indicators and sleep scores were often not clearly explained, leading to uncertainty about their meaning and accuracy. This lack of contextualisation sometimes contributed to strict self-evaluation, or distrust and disengagement, the latter particularly when numerical outputs appeared overly simplistic or inconsistent with lived experience. At the same time, smartwatch data were perceived as potentially useful for reflection, self-monitoring, and behavioural awareness, especially when integrated into therapeutic conversations. Importantly, lived experts emphasised the need for clear guidance on the meaning and purpose of smartwatch data.

Professionals described the treatment as a process where patients moved from dependency toward regaining their autonomy and structure in life. They explained that the use of the smartwatches was not yet part of their daily practice. While there was an installation manual, further guidelines were lacking: “We have too few guidelines to really do something with [the smartwatches]”, and professionals expressed a need for guidance on “why do we use [smartwatches] and what do we do with it?” In terms of format, the large majority of professionals (6 out of 8) preferred the card set, since they could easily pick it up during a treatment session to get some guidance.

When asked what they thought of compassion and its elements, professionals said that the elements of compassion are important for achieving treatment goals. One professional stated that the focus on compassion felt somewhat superfluous, since one would not quickly indicate that compassion is not important. Another professional, in another session, described compassion as “automatic, […] otherwise you cannot work here”. Out of the compassion elements, the awareness of the universality of suffering (simplified to ‘letting patients experience that others are going through similar things’) was seen as especially important during the group treatment according to professionals.

When asked explicitly what they thought of a focus within implementation materials on either efficient working due to rising healthcare costs versus gaining more insight into patients and offering better care, a professional responded: “I think for us, the care for the patient is the most important. If you talk about policymakers, they will probably find those healthcare costs very interesting”. Another professional stated: “I’m open to doing that [using smartwatches] and I do believe that it can yield something, but not because the ‘big guys’ make a lot of money from it”. The compassion symbols on the implementation materials were seen by some professionals as helpful and by others as unnecessary. Especially professionals who hesitated about the true reason behind the purchase of the smartwatches by their department seemed to appreciate the explicit attention for compassion. Overall, they valued the clarity and practicality of the ‘efficiency-focused’ prototypes, as well as the friendly, less coercive tone and attention for the personal process in the ‘compassion-focused’ prototypes.

### Requirements for implementation materials

Table 2 shows an overview of the needs and issues found in the co-design sessions and how they were translated to design requirements. These needs and issues could be divided into (1) treatment experiences and needs, (2) smartwatch experiences and needs, and (3) implementation materials needs and preferences.

**Table 2.** Overview of the needs and issues found in the co-design sessions and how they were translated to design requirements.

Need/Issue (Session(s) it was found in; identified by lived experts/professionals/both)	Translation to design requirement
Treatment experiences and needs	
It can be difficult for patients to recall and remember things that happened to discuss them during treatment. (1; lived experts)	1. Implementation materials should facilitate recall of patients’ previous experiences, and make them aware that certain types of information can be outsourced to a wearable.
The part-time treatment is experienced as a safety net with room for (difficult) feelings, which patients highly appreciate. (1; lived experts)	2. Implementation materials should give space for sharing (difficult) emotions, feelings, and experiences.
Patients can be hard on themselves in reaching (high) goals, such as the standard goal used by the wearable of 10.000 steps per day, which can be demotivating. (1; lived experts)	3. Attention should be paid to setting personal, helpful, and attainable goals instead of a standard (high) goal. Implementation materials should convey that the process of trying is more important than the pressure of reaching goals.
Home is not always an understanding and supportive environment, despite potential good intentions. (1; lived experts)	4. Implementation materials should help to transfer the support received in treatment to the home situation for patients.
If the patient is at home, there is typically no contact with them. The aim is that they can take what they learn in treatment and apply it at home. (2; professionals)	
Mental health staff rotates a lot, making it less clear what a patient has already been offered. (2, 4, 5; both)	5. Implementation materials need to be usable by rotating mental health professionals. It should be easy to know which cards have been discussed when, and to reflect with patients on topics that were discussed earlier.
Smartwatch experiences and needs	
It could be motivating to set goals (e.g. steps a day) together in the group. However, this should not be done in a competitive way. (1; lived experts)	6. Implementation materials should contribute to a supportive group environment, without competition.
The moment in treatment could be of importance when considering the smartwatch. At the start of treatment it could be too soon after a psychological crisis and be too much for a patient. In that case, the smartwatch could also only be used during one session instead of worn the whole time. (1,2; both)	7. In the implementation materials, there should be consideration for when to start the use of the smartwatches with the patient. It could also be indicated that a smartwatch can be used only during a specific session.
The smartwatch data (as presented on the watch and in the app) gives the impression of being precise. Patients find it frustrating and confusing if data does not seem correct. (1,5; lived experts)	8. Implementation materials should help to nuance the (necessity of) precision of the smartwatch data.
In a mental crisis, patients can be more sensitive to overstimulation: some patients find the amount of vibrations from the smartwatch too much and get annoyed by this. Professionals also indicate that it is important to support patients in turning off annoying social media notifications. (2; professionals)	9. Implementation materials should provide guidance for the professional in setting up the smartwatch in a suitable manner with the patient, e.g. turning off vibrations and notifications.
Professionals would like a course on the smartwatch and a very accessible, visual folder for patients. (2; professionals)	10. Implementation materials should come with a course and a patient folder.
It would be interesting to compare smartwatch data with subjective experiences, and see if these align or not. (2; professionals)	11. The implementation materials should pay attention to subjective experiences and comparison with smartwatch data.
Professionals preferred attention for fostering conversations over a focus on goals and precise numbers. (3; professionals)	
The materials should also address people who are not using a smartwatch. The group process needs to be protected. (4; professionals)	12. Implementation materials should be usable in a group where some patients wear a smartwatch and others do not, supporting an equal division of attention to all patients as much as possible.
Patients using smartwatches tend to elaborate more on their data and experiences than patients not using smartwatches. (5; lived experts)	
Patients miss support in working with the smartwatch and seeing its added value. (5; lived experts)	13. Implementation materials need to help in conveying the added value of smartwatches and interpreting their data.
Implementation materials needs and preferences	
The majority of professionals (6/8) preferred the format of a card set. They appreciated the tangibility of the card set, and being able to pick it up easily during a group session without much preparation or reading time. Two out of 8 participants preferred the format of a written manual. Four out of 8 participants indicated that they were interested in combining the card set with a written manual. (3; professionals)	14. Implementation materials should have the format of a tangible and easily accessible card set, potentially in combination with a written manual for background information.
Professionals want visual elements and colors on the implementation materials, but not too many. The materials should be playful but not childlike. They preferred text to be concise, concrete and clear, with bullet points and absence of difficult terms. (3,4; professionals)	15. Implementation materials should contain colors and visual elements that are playful but not patronizing. Text should be kept concise and accessible, with bullet points. Each card in the set should only contain one question, discussion topic, or exercise, instead of multiple.
The materials should also be usable by the patients themselves, and not just by the professional during the sessions. (3; professionals)	16. Implementation materials should provide the possibility for use by patients themselves.
It would be interesting to add challenges, to look at moments of relaxation and tension, heart rate variability (HRV), and the ‘body battery’ measurements of the smartwatch. (3; professionals)	17. Implementation cards should include challenges and the possibility to look at relaxation and tension, HRV and ‘body battery’ measurements.
Compassion symbols on prototypes were found helpful by some professionals, while the proposition of using the smartwatch to improve efficiency did not have added value for them, and the idea that this could be a motivation to use smartwatches led to resistance. Other professionals found the compassion elements recognizable, but not necessary on the prototypes. (3,4; professionals)	18. Implementation materials should contain compassion symbols, as they can be helpful for some professionals to show the compassion-focused rationale of using the cards and smartwatches, rather than merely for efficiency.

### Evaluation of the card-set prototype in session 5

After using the card set during a group treatment session, the professional who facilitated the session evaluated the prototype on several aspects. The card set received a mean score of 4 on the Compassionate Technology Scale for Professionals (CTS-P; maximum score = 5, SD = 0.59). The professional also rated the card set 4 out of 5 on the UMUX-Lite, the perceived usefulness items, and the item assessing added value derived from the System Usability Scale. Furthermore, the professional indicated they would be “somewhat disappointed” if the card set were no longer available (product-market fit item) and rated the likelihood of recommending the card set to colleagues as 7 out of 10.

### Final design: A compassion-focused card set for reframing the integration of wearables into treatment

Based on the requirements collected during the co-design sessions, and with the help of a design company, we developed implementation materials in the form of a compassion-focused card set (see Figure 8). Almost all requirements could be implemented in the final design of the card set (see Table 3). The set consists of big ‘statement cards’ in three categories: blue for sleep and relaxation, orange for activity, and green for reflection. These cards start with a statement, for example “I go to bed and get up at the same time every day”. The mental health professional can read this statement to the group, and patients can raise their hand if this statement applies to them. In the case of the example statement, if only few people raise their hand, the card is relevant for the group. Below the statement, there is a description of a discussion topic or small exercise that relates to the statement and can be discussed or tried out in the session. On the backside of the card, there is a brief explanation of how the smartwatch or the smartwatch phone application can help provide input for the conversation or support with the exercise.

**Figure 8.**
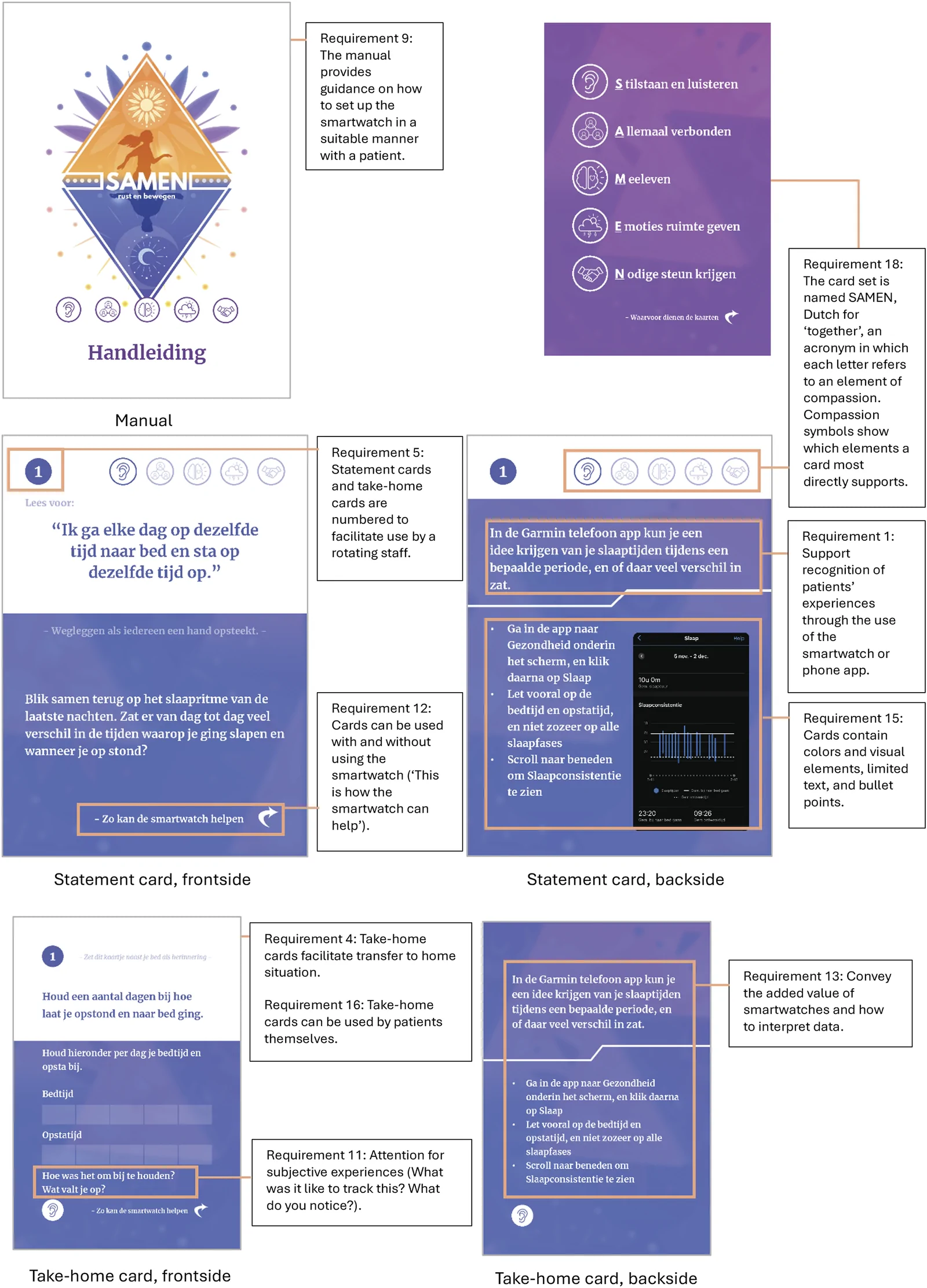
Impression of the final card set design, with examples of how requirements were implemented. *Note: the design is shown in its original, Dutch language*.

**Table 3.** Implementation of design requirements into the final design. If (part of) a requirement was not met in the final design, it is shown in italic.

1. The backsides of the statement cards and take-home cards show how the smartwatch can help gain insight into sleep and activity patterns.
2. Through the compassion symbols on all cards, users are reminded of compassion elements such as openness to difficult emotions (distress tolerance). The text on cards stimulates to have attention for difficult experiences and the process rather than outcomes.
3. In the manual and on a card specifically referring to goal setting, there is emphasis on setting a personal and attainable goal and experiencing if that goal is suitable. A standard goal of 10.000 steps is not recommended.
4. The take-home cards contain information and exercises to do at home, to facilitate transfer from treatment to the home situation and offer a form of support.
5. All cards are numbered, with numbers on take-home cards corresponding to those on the matching statement card.
6. Text on the cards and in the manual emphasizes that goals and challenges are personal, and not meant as a competition. Where relevant, cards focus on letting patients share supportive information with each other, such as tips.
7. Where relevant, statement cards indicate that a smartwatch could be used only temporarily for that card, instead of consistently. *The implementation materials currently do not provide information on when in treatment it is best to start the use of a smartwatch with patients, as there is not yet enough experience or research on this topic*.
8. The cards and the manual emphasize that the smartwatch data is not always completely accurate or precise, and how the smartwatch may still be helpful despite this (e.g. by looking at sleep consistency over time rather than sleep phases).
9. The manual contains detailed information and screenshots on how to personalize the smartwatch settings, e.g. how to turn off vibrations and social media notifications.
10. In a follow-up study to the current one, professionals received a short training to familiarize themselves with the implementation materials. *We did not provide a course on the use of the smartwatch or a folder for patients, as this was outside the scope of the current study*.
11. All cards have a focus on the process of the patient and their subjective experiences, if relevant in comparison to the smartwatch data.
12. Cards were designed to be relevant for patients who wear the smartwatch and patients who do not. The front-sides of all cards apply to all patients. On the back-side of the cards, information or exercises can be found on how the smartwatch can help. Statement cards contain statements to check their relevance for the group, and to involve all patients as much as possible.
13. The take-home cards for patients contain information on the added value of the smartwatches and if relevant, basic information on how to interpret their data.
14. The format of a card set in combination with a written manual was chosen.
15. The implementation materials contain colors and some visual elements in the form of compassion symbols and screenshots. Text is kept concise and accessible, and where possible, bullet points are used. Each card only contains one question, discussion topic, or exercise.
16. The take-home cards can be used by patients themselves outside of the sessions.
17. There are specific ‘challenge’ cards and cards about tension and relaxation. *However, HRV and ‘body battery’ measurements were not included due to lack of scientific studies on the validity of these measurements in ambulatory contexts*.
18. Each card contains the compassion symbols that the card can help support. In the manual, attention is given to the meaning of compassion and its elements, and that compassion is the cornerstone of the card set and the use of the smartwatch. The set of implementation materials is called SAMEN, a Dutch acronym meaning ‘together’, in which each letter refers to one element of compassion.

Each big statement card comes with a corresponding and smaller ‘take-home’ card. These take-home cards contain exercises that match with the topic of the big statement card that is used in the treatment session. The idea is that after having discussed a card in the session, patients can take home a small card to work with, for example to keep track of sleep or reflect on their activities at home. The green reflection card can be used by the professional to reflect with the group on the take-home cards that patients have worked with, and prompts them to focus on the experiences and processes of patients rather than whether everything worked out perfectly. All cards contain symbols referring to the five compassion elements proposed by Strauss et al.^28^ These symbols indicate to the professional (in case of the big statement cards) and patient (in case of the take-home cards) which elements of compassion the use of the card (and potentially the smartwatch) can contribute to. For example, discussing and tracking sleep consistency can help to get more insight into sleep behavior and as such help to recognize issues that cause suffering.

The card set comes with a quick guide and a manual. The quick guide describes the parts of the card set, explains the compassion-focused rationale behind the set and the compassion symbols, and helps the professional to quickly start using the cards. The manual gives guidance on how the smartwatch and the smartphone app can be set up, as well as some points of attention when using the smartwatch with patients.

## Discussion

This study explored the process of the value-based co-design of implementation materials for the use of smartwatches in mental healthcare, with a focus on the value of compassion. We will discuss the requirements we found and the main considerations for the final design of a compassion-focused card set. Furthermore, we will reflect on how value-based co-design can contribute to meaningful and effective implementation strategies.

### Requirements for smartwatch implementation materials and considerations for the final design

In the context of the current study, professionals were already offered clear and detailed information on how to install smartwatches for patients before the study started. However, they indicated needing more support in how and why to use the smartwatches in treatments. This finding confirms earlier studies describing a lack of attention in literature on digital mental health interventions for how they could be combined with regular treatments,^29^ and the requests from professionals to receive clear motivation and guidance to integrate digital tools in treatment.^18,33^ Lived experts similarly emphasised the need for support in the interpretation of smartwatch data, particularly regarding stress indicators and sleep metrics, and how these relate to their subjective experiences. They also highlighted the importance of guidance to understand the added value and limitations of smartwatch use. These findings confirm earlier research that stressed the need to provide guidance to patients who use smartwatches.^16^ The current study was aimed at narrowing this gap by exploring the co-design process of implementation materials for the meaningful integration of an existing technology.

The majority of participating professionals preferred implementation materials in the form of a card set, rather than an infographic or written manual. They appreciated that a card set contained to the point information and support that they could easily pick up and use during a treatment session. All professionals disliked the infographic, since there were already too many infographics in use in the mental healthcare context according to them. The written manual was generally not preferred, which is in line with earlier literature indicating that a rigid protocol might not (always) be fitting for the use of digital tools in specialized mental healthcare, due to the complexity of problems encountered,^20^ and the preference for professional autonomy.^17^ Moreover, professionals in the current study wanted the implementation materials to help protect the group process in treatment sessions, by not excluding patients who do not want to use a smartwatch. This points to the need of being able to use guidelines in a flexible way, including leaving open the option for patients not to use technology.^3^

Regarding the framing used in the prototypes, both professionals and lived experts expressed the importance of implementation materials facilitating attention for individual processes and experiences, including giving space for sharing difficult emotions and experiences, rather than a focus on quantitative goals and outcomes. Mental healthcare professionals have previously expressed concerns about patients focusing too heavily on data when using wearables, as this could have negative effects.^16^ Fears exist that the use of wearables might only be suitable for or appealing to “fitness fanatics”, and that they could be demotivating due to failure to achieve goals.^31^ Given that most wearables where indeed originally designed for commercial fitness purposes,^51^ implementation materials can play an important role in their conscious use in mental healthcare. The accounts of the participating lived experts confirm that smartwatch data could be experienced as both supportive and stressful, depending on how it is introduced and contextualised within treatment. Many lived experts emphasised that without sufficient explanation, monitoring could reinforce self-critical responses or uncertainty. Having compassion as an explicit cornerstone in the use of smartwatches can help to build a holistic picture of each patient rather than getting lost in quantified data and achievements, for example by setting attainable goals and having attention for the personal process. Lived experts expressed the importance of feeling seen, understood, and supported in the treatment process and when using digital tools.

However, in response to compassion-focused and efficiency-focused prototype styles, professionals appreciated elements of both. On the one hand, they preferred text to be to-the-point and practical, as found in efficiency-focused prototypes. On the other hand, they expressed that a compassionate tone with attention for subjective experiences matched their work with patients better. Some professionals were more indifferent to the use of compassion symbols as they saw those as obvious in their work, while more technology-critical professionals preferred them and explicitly rejected the efficiency narrative used in some prototypes.

Importantly, the interpretation of these findings should be considered in light of the study framing. Compassion was embedded throughout the study design, materials, and researcher positioning, which may have introduced social desirability effects, with participants potentially aligning responses with perceived expectations around compassionate and person-centred care. At the same time, the co-design process also included efficiency-oriented prototypes, which were not explicitly labelled as such to avoid priming effects. This allowed participants to engage with the concrete design elements rather than predefined categories. Participants nevertheless spontaneously articulated concerns about an overemphasis on efficiency in the use of technologies such as smartwatches, while also expressing nuanced preferences for certain efficiency-oriented features. This suggests that participants were able to critically and flexibly engage with both framings, although their responses were likely shaped by the broader compassionate orientation of the study. Based on previous research on the importance of compassion in mental healthcare^23–26^ and our current findings, we developed compassion-focused implementation materials, but also incorporated the more to-the-point style of the efficiency-focused prototypes.

The final, tangible card set design is meant as a bridge between two worlds: the ‘objective’ measuring capabilities of smartwatches and the personal, talk-based approach of regular mental health treatment. The card set design aligns with the concept of ‘compassionate technology’ where mental health professionals and technology complement each other to offer compassionate and sustainable care.^29^ The format of a card set does lead to an interesting paradox: while digital innovations such as internet and mobile interventions have the merit of being highly scalable,^52^ we have developed a physical, non-technological card set that does not provide that advantage to the same extent. A mobile phone application with the same contents as our card set, for instance, would have been much more scalable and adaptable. However, it would also face the risk of being ‘invisible’ in one’s phone, and moreover, just another piece of technology that one may be hesitant to use in a treatment session.^53^ In contrast, as stated before, the tangibility and physical presence of the card set during treatment sessions was highly preferred by participating professionals, and could also serve as a visible reminder to be picked up, used, and to explore the potential of using the smartwatches. Therefore, although the card set is typically introduced by a therapist or facilitator, it can also be used independently. Clients can take the cards home, enabling continued reflection and experimentation outside of scheduled sessions. This extends the intervention’s reach in both time and context, offering a form of scalability without requiring ongoing human presence.

### The role of value-based co-design in implementation processes

In general, taking on a participatory approach towards implementation processes through the active involvement of stakeholders is highly recommended.^54,55^ Taking this further, the current study shows how a co-design process that is sensitive to contextual values can be helpful in bringing not just surface-level preferences but also underlying concerns and convictions of stakeholders to the surface. In this case, it triggered participants to reflect on and share their beliefs around technology use, aided by co-design materials that had efficiency-focused or compassion-focused narratives. Such insights about the values held by stakeholders are crucial to understand and respond to deeper processes behind the (non-)adoption of technologies in healthcare.^56^

For example, during the current co-design process, it became clear that participating professionals were not necessarily opposed to the use of technology in mental healthcare. However, some of them expressed doubts about the deeper motivations behind the introduction of technology in treatments, as well as the consequences technology use could have. Such doubts and fears revolved around whether technology really improves care or worsens it, whether it is actually acquired for financial reasons rather than care quality, and technology being too solution focused, lowering our distress tolerance as humans. These and similar sentiments were also found in previous research using interviews with mental healthcare professionals.^15,17,57^ Professionals that expressed more doubts about technology use in mental healthcare seemed to have a stronger preference for compassion as motivation to use technology, and the presence of concrete compassion symbols. Therefore, the explicit compassion-focus could be useful in providing hesitant professionals with the needed meaningful conceptual basis of technology use that previous research called for.^18^

### Strengths and limitations

A major strength of the current study was the integration of initial contextual inquiry and participatory co-design, ensuring that both contextual understanding and stakeholder perspectives shaped the development of the intervention. Though our main focus was on supporting professionals with implementation materials, we could also get important insights from the experiences of lived experts, clarifying which types of guidance were needed for them in the use of smartwatches. During the co-design process we employed multiple iterative rounds to constantly evaluate and reiterate the prototypes and design choices, as is recommended.^54^ Furthermore, we were able to involve a design company to achieve a more attractive and well thought out final design. Finally, this study had a unique focus on compassion as essential value in mental healthcare. The value-sensitive approach helped to achieve a more in-depth co-design process, with attention for underlying needs and beliefs. Importantly, this did not remain at the level of abstract principles: the outcome of the project offers a concrete operationalization of compassionate care, both in form (a physical card set designed for therapeutic use) and in content (instructions and prompts that align with the five elements of compassion as proposed by Strauss et al.^28^). This contributes to bridging the gap between conceptual values and practical implementation strategies in digital mental health innovation.

This study also had limitations. Since our co-design study was conducted in a specific acute mental healthcare setting and involved a relatively small sample of professionals and lived experts, the findings may not readily generalize to other mental healthcare departments or stakeholder groups. In line with the pragmatic and real-world nature of the co-design process, participants were included based on availability rather than purposive stratification. As a result, the sample was heterogeneous in terms of both lived expert characteristics and professional roles. While this limits the ability to draw subgroup-specific interpretations, it did enable the inclusion of a broad range of perspectives that are highly relevant to real-world implementation.

Furthermore, due to time constraints, we had to be pragmatic with the number of co-design sessions that were feasible. In addition, the participating professionals naturally had busy schedules, so that the sessions could not be longer than 90 minutes. In other circumstances, we could have conducted more or longer sessions to provide space for a wider range of creative co-design exercises. For example, we could have made use of more time-consuming generative techniques where participants create artefacts in response to current challenges and to explore future scenarios, which can clarify their latent needs and wishes.^58^ Currently, we built three different formats of prototypes based on the first two co-design sessions, but in a more extensive co-design process the format could have been more open, which could have led to a different final design. Nevertheless, Larkin et al. state that it might be unrealistic to expect participants in co-design sessions to provide solutions to complex issues in a limited amount of time, advocating for a pragmatic approach.^59^ Moreover, we do believe that we tapped into more overarching design requirements that would also have arisen if we had used a different approach, as several requirements were mentioned by different participants and in different sessions. In addition, while we involved lived experts in the first and final co-design session, their role could have been more intensive. However, we deliberately chose to primarily concentrate on professionals and offering them clearer guidance for the use of technology, since earlier studies have shown that this is lacking.^18,29,33^

Finally, the current study did not include an empirical evaluation of the implementation materials in terms of their impact on actual smartwatch use in clinical practice. The present work was explicitly focused on the co-design and development of value-based implementation materials rather than their effectiveness. As such, conclusions are limited to the design process and stakeholder perspectives.

### Future research

The current co-design study has provided useful insights on the use and implementation of smartwatches in mental healthcare. A logical follow-up step is to systematically evaluate the use of the developed implementation materials in daily practice. A mixed-methods single case study with that purpose is currently being conducted to explore the influence of the compassion-focused card set on the use of smartwatches in mental healthcare, as well as their acceptance, perceived added value and perceived contribution to compassionate care by mental health professionals.

Further studies could also employ additional co-design sessions to expand the developed implementation materials so that they apply to all kinds of wearables and across different settings of mental healthcare, and not just to the type of smartwatch and setting in the current study. These studies could benefit from purposive sampling of more homogeneous stakeholder subgroups to allow for more in-depth exploration of specific perspectives. Moreover, we currently focused on a rather ‘simple’ application of smartwatches for keeping track of and adjusting lifestyle factors, as the validity of more complex health scores remains unclear.^60^ If more complex health scores are validated and the application of wearables for passive sensing becomes more common in current mental healthcare, future research could investigate what guidance is needed to support its use in daily practice.

### Conclusions

Our study shows how co-design could contribute to the meaningful integration of promising technologies in mental healthcare. Through co-design sessions with professionals and lived experts, we developed implementation materials to support professionals in embedding smartwatches in treatment. We found that professionals prefer a tangible format for implementation materials, which they can easily pick up during a treatment session. Moreover, we explored two different framings in the implementation materials: one that was efficiency-focused and one that was compassion-focused. Professionals and lived experts valued elements of compassion in treatments highly. An explicit focus on compassion in implementation materials seemed to be especially important to professionals who had doubts about using technology. As a final product, we were able to develop a compassion-focused card set with the help of a design company. By involving professionals and lived experts and foregrounding compassion, we developed implementation materials that help bridge the gap between conceptual values and practical strategies in blended mental healthcare.

## Supplemental Material

Supplemental Material - Reframing wearables in therapy: Co-designing compassion-focused, practical guidance for the use of wearables in acute mental healthcare Supplemental Material for Reframing wearables in therapy: Co-designing compassion-focused, practical guidance for the use of wearables in acute mental healthcare by Charlotte M. van Lotringen, Saskia M. Kelders, Gerben J. Westerhof, Matthijs L. Noordzij in Digital Health

Supplemental Material - Reframing wearables in therapy: Co-designing compassion-focused, practical guidance for the use of wearables in acute mental healthcare Supplemental Material for Reframing wearables in therapy: Co-designing compassion-focused, practical guidance for the use of wearables in acute mental healthcare by Charlotte M. van Lotringen, Saskia M. Kelders, Gerben J. Westerhof, Matthijs L. Noordzij in Digital Health

Supplemental Material - Reframing wearables in therapy: Co-designing compassion-focused, practical guidance for the use of wearables in acute mental healthcare Supplemental Material for Reframing wearables in therapy: Co-designing compassion-focused, practical guidance for the use of wearables in acute mental healthcare by Charlotte M. van Lotringen, Saskia M. Kelders, Gerben J. Westerhof, Matthijs L. Noordzij in Digital Health

## Data Availability

The data that support the findings of this study are available from the corresponding author, CvL, upon reasonable request. The data are not publicly available to protect the privacy of research participants.*
