# Supplementary material for: Reframing wearables in therapy: Co-designing compassion-focused, practical guidance for the use of wearables in acute mental healthcare
Source: Digit Health. 2026 Sep 22;12:20552076261483463. doi: 10.1177/20552076261483463 (PMC13748783; doi:10.1177/20552076261483463)
Supplement: Supplementary file 3 [file sj-pdf-3-dhj-10.1177_20552076261483463.pdf]

Compassionate Technology Scale for Professionals (CTS-P) (van Lotringen et al., 2024)

The statements below are about the extent to which a digital mental health intervention is supportive of various aspects of treatment. The wording ‘helps me to’ refers to whether the intervention contributes to this, not to the intervention being necessary for this.

Using the smartwatch and the supporting materials help me to…

	Completely disagree	Disagree	Neutral	Agree	Completely agree
Support a client in working towards their goals.	O	O	O	O	O
Accept difficult feelings that a client evokes in me.	O	O	O	O	O
Put myself in the perspective of a client.	O	O	O	O	O
Not look down on a client’s suffering.	O	O	O	O	O
Deal with my own difficult feelings that arise from empathizing with a client.	O	O	O	O	O
Recognize a client’s suffering.	O	O	O	O	O
Empathize with a client.	O	O	O	O	O
Realize that I could also be in a client’s situation.	O	O	O	O	O
Be aware of a client’s behavior.	O	O	O	O	O
Realize that having problems is human.	O	O	O	O	O
Alleviate a client’s suffering.	O	O	O	O	O
Get a good sense of a client’s thoughts and feelings.	O	O	O	O	O
Understand a client’s problems.	O	O	O	O	O
Support a client in improving their situation.	O	O	O	O	O
Prevent becoming overwhelmed with a client’s difficult emotions.	O	O	O	O	O

The questions below concern the card set.

	Completely	Disagree	Neutral	Agree	Completely

	disagree				disagree
The possibilities of the card set meet my requirements.	O	O	O	O	O
The card set is easy to use.	O	O	O	O	O
The card set can make it easier for me to do my job.	O	O	O	O	O
I plan to use the card set regularly.	O	O	O	O	O
The card set is of added value in the treatment.	O	O	O	O	O

	Very disappointed	Somewhat disappointed	Not disappointed
How would you feel if you could no longer use the card set?	O	O	O

	Very unlikely									Very likely
How likely are you to recommend the card set to your colleagues?	1 O	2 O	3 O	4 O	5 O	6 O	7 O	8 O	9 O	10 O
